# Ultra-broadband MIR super absorber using all silicon metasurface of triangular doped nanoprisms

**DOI:** 10.1038/s41598-022-18817-1

**Published:** 2022-08-31

**Authors:** Mostafa Abdelsalam, Mohamed A. Swillam

**Affiliations:** Department of Physics, School of Sciences and Engineering, The American University in Cairo, New Cairo, Egypt

**Keywords:** Metamaterials, Silicon photonics

## Abstract

Thermo-electric generation offers to be a solid candidate for both dealing with the temperature problems of photo-voltaic cells and increasing its total output power. However, it requires an efficient broadband absorber to harness the power found in the near and mid-infrared regions. In this work, we discuss a new structure of nanoprisms that are made of doped silicon that acts as an ultra-broadband absorber in both regions. We also discuss the effect of the doping concentration. Additionally, we study the effect of a pure silicon thin film on top of the prisms. Finally, we’re able to find an optimized structure that can absorb 92.6% of the input power from 1 to ${15}\,{\upmu }$m.

## Introduction

The demand for a sustainable source of energy is increasing by the day. One of the most popular candidates is solar energy. Photo-voltaic solar cells can be used to absorb some of the power in the visible range^1^. However, the photon energy in the near infrared wavelengths (NIR) is not high enough to to produce free electron–hole pairs that can be used as a source for electrical power^2^. In fact photons that have such low energy can have negative side effects when absorbed as they are turned into heat energy increasing the temperature and in turn decreasing the efficiency photo-voltaic cell^3^.

To solve this problem thermo-elecric generation (TEG) uses the heat energy produced by the NIR photons to generate electricity of its own^4–6^. This requires a temperature gradient between a cold and hot surface. Producing the hot surface can be made using a NIR absorber that can absorb the NIR wavelengths in the solar spectrum (1–${4}\,{\upmu }$m)^7–9^. Additionally, this technique can be used to produce electricity even in the night using Earth’s black body radiation^10^. However, Earth’s black body radiation is in the mid infrared (MIR) range. Thus, it is required to find an ultra-broadband absorber that can cover both the NIR and the first half of the MIR range (i.e. roughly from 1 to ${15}\,{\upmu }$m). Usually, most absorbers designed for TEG is designed to absorb only one of the two ranges. Absorbers can also be used for thermal photo-detection which also requires broadband operation that can be tailored to specific applications.

Additionally, usually these structures depend on the plasmonic effects of noble metals like silver and gold^11,12^. These metals have high cost plus they are not CMOS compatible which adds complexity to on-chip fabrication. This also rules out its use for thermal photo-detection. Heavily doped Silicon proves to be a good candidate for replacing noble metals^13–17^. On top of it being CMOS compatible, its doping can be engineered to tailor the absorption spectrum to the required bandwidth. Using the Drude model one can manipulate the plasma frequency of doped Silicon and in turn tailor the absorption spectrum of the absorber.

In this work we will show the effect of texturing a highly doped silicon layer on a silicon substrate on the optical power absorption efficiency in the NIR and MIR waves. The texturing will consist of periodic triangular nanoprisms. We will also study the effect of changing the dimensions of the prism on the absorption spectrum. Additionally, we will see the effect of having a thin film coating of pure silicon on top of the structure on the absorption efficiency.

## Methods

**Figure 1:**
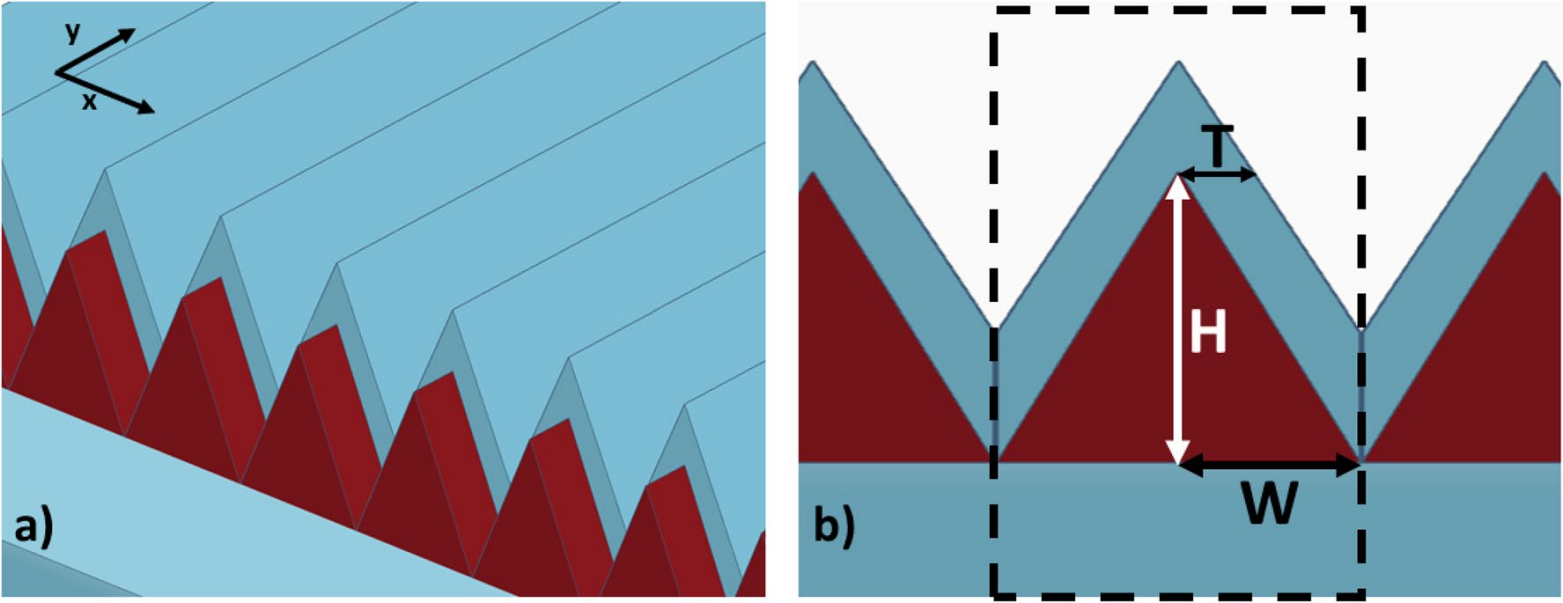
(**a**) 3D schematic of the structure showing doped silicon triangular nanoprisms in red and silicon substrate and coating in blue, (**b**) cross-section of the structure showing a unit cell in the dashed boundary.

The structure depends on texturing the surface of heavily doped silicon then coating the structure with a specific thickness of pure silicon. The surface texturing is an infinitely long triangular nanoprism of highly doped silicon on top of a silicon substrate. This prism facilitate the coupling of the plane wave from free space to the structure. We study the structure with and without silicon coating to increase the absorption bandwidth in Fig. 1a. This absorbed power is then converted to heat which can be used for thermo-electric generation.

This periodic structure have three parameters that can be engineered to reach the required absorption spectrum (Fig. 1b). These parameters are the height of the doped-silicon nanoprism (H), its base (2W), the horizontal thickness of the silicon coating (T), and the spacing between the doped-silicon nanoprism to the end of the unit cell of the periodic structure. We use Finite-Difference Time Domain (FDTD) to simulate the absorption spectrum. This method divides the simulation regions to multiple small meshes, which inherently add some inaccuracies, to simulate the behaviour of the wave inside the structure. We then sweep over the three parameters to further increase the absorption bandwidth in the infrared wavelength. We try to increase the absorption from 1 to ${15}\,{\upmu }$m to be able to harvest both the solar energy in the NIR domain and the radiation from earth’s black body radiation.

We use the data found by Palik et al.^18^ for the optical parameters of pure silicon in the IR domain. As for the doped Silicon will be modeled using Drude model that evaluates the permittivity of doped material as follows^19^:

1 $$\begin{aligned} \varepsilon \left( \omega \right) = \varepsilon _{\infty }-\frac{\omega _{p}^{2}}{\omega ^{2}+j\omega \Gamma } \end{aligned}$$

where $\varepsilon \left( \omega \right)$ is the permittivity of the doped silicon as a function of the angular frequency $\omega$, $\varepsilon _{\infty }$ is the high-frequency permittivity limit for silicon which is 11.7, $\Gamma$ is the scattering rate which can be defined as

2 $$\begin{aligned} \Gamma = \frac{q}{m^{*}\mu } \end{aligned}$$

where *q* is the electron charge, $m^{*}$ and $\mu$ the effective mass,which is equal to 0.26 of the electron mass ($m _e$), and mobility of the free carrier respectively, and finally, $\omega _{p}$ is the plasma frequency which is defined as:

3 $$\begin{aligned} \omega _p = \sqrt{\frac{N _d q^{2}}{\epsilon _{0} m^{*}}} \end{aligned}$$

where $N _d$ is the doping concentration, and $\epsilon _{0}$ is the permittivity of free space. This allows for doped silicon to be tailored for a specific absorption range. Since we’re focusing on thermal harvesting application, we’ll be only studying one doping concentration that focuses on the NIR and MIR applications. However, this can be tailored for different wavelengths by changing both the doping and dimensions of the structure.

**Figure 2:**
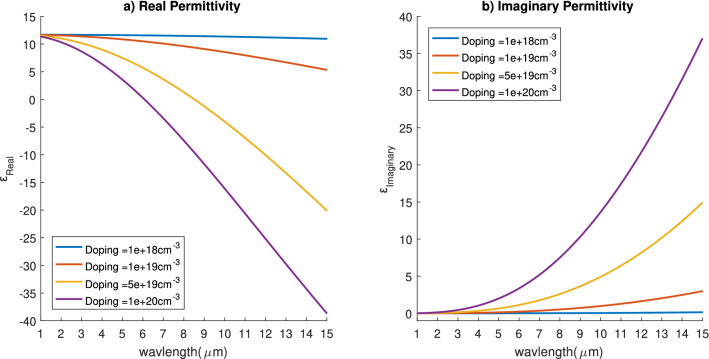
(**a**) Real and (**b**) imaginary parts of the permittivity of silicon at different doping concentrations.

When applying Drude model for different doping concentration of Phosphorus we get the real and imaginary parts of the permittivity as shown in Fig. 2. The figure shows that as we increase the doping concentrations the imaginary part of the permittivity increases. On the other hand, the real part of the permittivity decreases as we increase the doping concentration reaching negative values. This indicates that as the doping concentration increases, the doped silicon’s losses increases. These losses are due to the free electrons in highly doped silicon which leads to absorbing the incident electromagnetic wave and converting its power to thermal energy. This can be used to increase the thermal power absorbed from the solar spectrum for thermo-electric generation applications. Throughout this work, we will be measuring the absorbed power normalized to the incident power. This normalized power is calculated as

4 $$\begin{aligned} {P_{abs}(f)}= {1-} { T(f)-R(f)} \end{aligned}$$

where

5 $$\begin{aligned} {V(f)}= \frac{1}{2 {{P_{inc}}}} {\int _{-w}^{w}\mathfrak {R}e( {P(f)) \cdot dS_v}} \end{aligned}$$

where; *V*(*f*) is either the normalized transmission *T*(*f*) or reflection *R*(*f*) as a function of frequency, *P*(*f*) is the Poynting vector, *w* is half the width of the unit cell as clear in Fig. 1, and ${dS_v}$ is the normal of the surface either for the transmission port ($S_T$), or the reflection port (${S_{R}}$).

## Results and discussion

**Figure 3:**
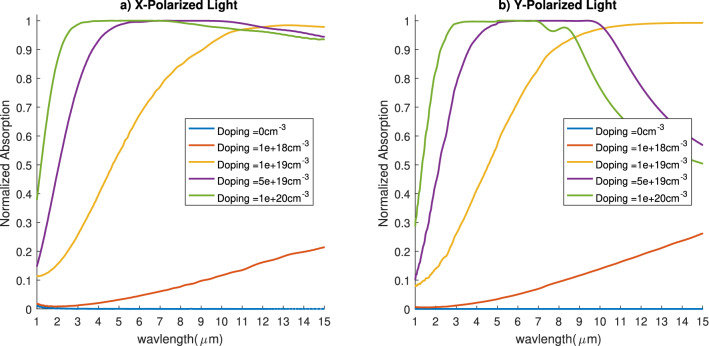
Absorption spectrum for different doping concentrations for x-polarized waves (**a**) and y-polarized waves (**b**).

First, we discuss the effect of the doping concentration on the absorbed spectrum (Fig. 3) silicon nanoprisms without any coating. This was studied for nanoprism height of ${20}\, {\upmu }$m and width of ${5}\, {\upmu }$m (i.e. H = ${20}\, {\upmu }$m and W = ${5}\, {\upmu }$m). As expected, pure silicon doesn’t absorb any of the power in the IR domain. However, when the doping concentration is increased, we see a drastic increase in the absorbed power. As shown in Fig. 3, a doping concentration of $10^{20}\,{\text {cm}}^{-3}$ has the highest absorption in the NIR domain for both the x and y-polarized electromagnetic wave. However, in the MIR domain we can see that there is a drop in the absorbed power as the doping increase. The discrepancy between both polarization is due to the fact that the structure is not symmetrical in both directions. It can be also attributed to the increased number of highly doped (metal-like)/dielectric interfaces along the x direction more than the y direction. These interfaces help in localizing the optical field withing the highly doped layer. The scope of this work is to design an ultra-broadband absorber for solar thermal energy harvesting which is mainly in the NIR region. That’s why we’ll focus from now on studying silicon with doping concentration of $10^{20}\,{\text {cm}}^{-3}$.

**Figure 4:**
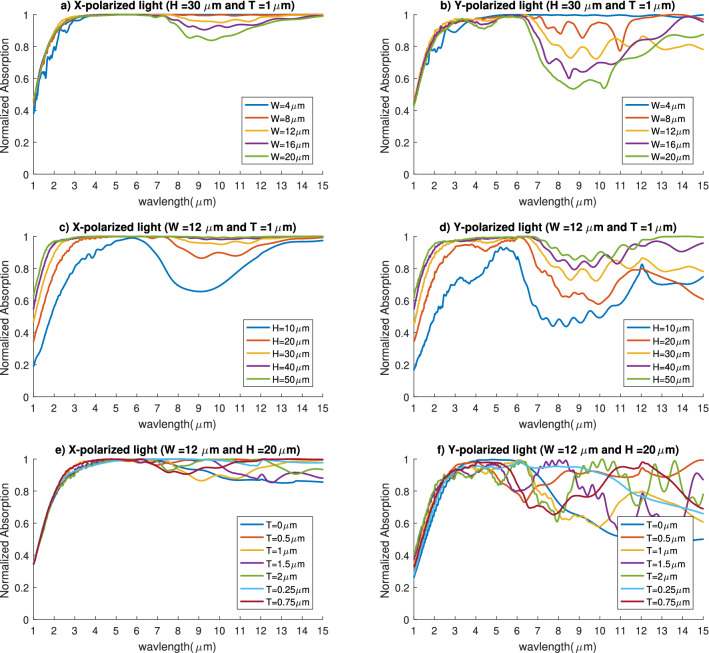
Absorption spectrum for different nanoprism dimensions. (**a**) Absorption of x-polarized waves for different widths, (**b**) Absorption of y-polarized waves for different widths, (**c**) Absorption of x-polarized waves for different heights, (**d**) Absorption of y-polarized waves for different heights, (**e**) Absorption of x-polarized waves for different silicon coating thickness, (**f**) Absorption of y-polarized waves for different silicon coating thickness.

Keeping the doping concentration at $10^{20}\,{\text {cm}}^{-3}$, we made several sweeps to maximize the absorbed power in the IR domain. We started by keeping the height and silicon coating thickness constant at ${30}\,{\upmu }$m and ${1}\,{\upmu }$m, respectively. Figure 4a,b show the absorption spectrum for multiple prism widths both for the x and y-polarized waves. There is a slight decrease in the MIR region for the x-polarized absorption as the width. On the other hand, for y-polarized light the decrease is much more noticeable.

On top of that, if we kept the prism width and the silicon coating thickness constant at ${12}\,{\upmu }$m and ${1}\,{\upmu }$m while changing just the height, we can see that the height affects the absorption across the whole region of study (Fig. 4c,d). The absorption suffers a lot at small heights while excels at very large heights. The absorber can achieve near-perfect absorption across the whole study region for the x-polarized light except for a small region in the NIR. As for the y-polarized light there is a slight drop in the absorption curve in the MIR domain, but it still follows the same trend as the x-polarized light absorption. Keeping that in mind we can conclude that the steeper the slope of the prisms, the higher the absorbed power. Specially in the NIR region we can see that the slope of the nanoprisms greatly affect the normalized absorption. We can also see that the y-polarized absorption suffers more than that of the x-polarized light if we decrease the slope of the prisms.

Additionally, when we keep the prism width and height at ${12}\, {\upmu }$m and ${20}\, {\upmu }$m and only change the silicon coating thickness, we couldn’t find a specific behavior for the absorption spectrum (Fig. 4e,f). However, we can see that it can affect the absorption for both polarization. So we did an optimization on the silicon coating thickness to find the dimensions that achieve the highest absorption. Here we study the highest absorption in the NIR (from 1 to ${4}\, {\upmu }$m) and the MIR (from 4 to ${15}\, {\upmu }$m) regions separately. This allows us to find the different dimensions that achieve the highest absorption in each region for both polarization.

**Figure 5:**
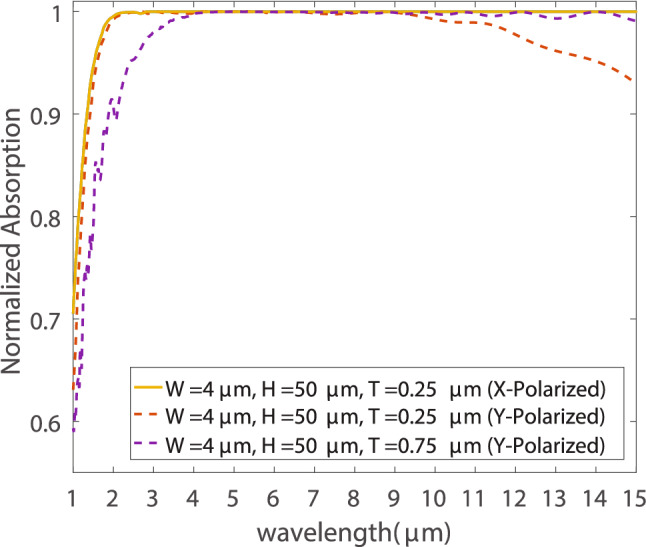
Absorption spectrum for the nanoprisms achieving maximum absorption for x-polarized waves (solid lines) and y-polarized waves (dashed lines).

For x-polarized light, we found that the same set of dimensions achieve the maximum absorption in both the NIR and MIR regions. As shown in Fig. 5, at width of ${4}\, {\upmu }$m, height of ${50}\, {\upmu }$m, and silicon coating thickness of ${0.25}\, {\upmu }$m, the structure can absorb 92% of the incident x-polarized light in the NIR and almost 100% of the total MIR x-polarized light. As for the y-polarized light, there appears to be a trade-off between the absorption in the NIR and MIR. The previously mentioned dimensions also have the highest absorption in the NIR for y-polarized light, absorbing 89.65% of the incident power. However, in the MIR another set of dimensions achieve the highest absorption which is 99% of the y-polarized waves in the MIR. The width is ${4}\, {\upmu }$m, height is ${50}\, {\upmu }$m, and the silicon coating thickness is ${0.75}\, {\upmu }$m, which is the only parameter that differs from the previously mentioned dimensions. Finally, since we’re using this for solar thermo-electric generation we calculate the total power absorbed from both polarization (x and y) in the whole study region from 1 to ${15}\, {\upmu }$m. We found that the first set of dimensions are able to absorb 92.6% of the total incident power which is the maximum total absorption we could achieve. It is worth noting that, the minimum spatial step size utilized in the FDTD simulations was 20 nm. Reducing the step size further by 10% has an impact of changing the results with less than 1% for all the studies cases.

## Conclusion

In this work we studied a novel nanoprism structure made of highly doped silicon as an ultra-broadband absorber. We studied the effect of the doping concentration, prism height, width and the thickness of a thin pure silicon coating on the absorption spectrum. We found multiple optimized structures for different polarization conditions and different wavelength ranges. In the end, we were able to achieve maximum absorbed power of 92.6% of the total incident power on the absorber between 1 and ${15}\, {\upmu }$m.

## Data Availability

All data needed to evaluate the conclusions in the paper are present in the paper.
